# Microbial communities affecting albumen photography heritage: a methodological survey

**DOI:** 10.1038/srep20810

**Published:** 2016-02-11

**Authors:** Andrea Puškárová, Mária Bučková, Božena Habalová, Lucia Kraková, Alena Maková, Domenico Pangallo

**Affiliations:** 1 Institute of Molecular Biology, Slovak Academy of Sciences, Dúbravská cesta 21, 84551 Bratislava, Slovakia; 2 Slovak National Archives, Department of Archival Preservation, Drotárska cesta 42, 84005 Bratislava, Slovakia; 3 Caravella, Ltd., Tupolevova 2, 85101 Bratislava, Slovakia

## Abstract

This study is one of the few investigations which analyze albumen prints, perhaps the most important photographic heritage of the late 19^th^ and early 20^th^ centuries. The chemical composition of photographic samples was assessed using Fourier-transform infrared spectroscopy and X-ray fluorescence. These two non-invasive techniques revealed the complex nature of albumen prints, which are composed of a mixture of proteins, cellulose and salts. Microbial sampling was performed using cellulose nitrate membranes which also permitted the trapped microflora to be observed with a scanning electron microscope. Microbial analysis was performed using the combination of culture-dependent (cultivation in different media, including one 3% NaCl) and culture-independent (bacterial and fungal cloning and sequencing) approaches. The isolated microorganisms were screened for their lipolytic, proteolytic, cellulolytic, catalase and peroxidase activities. The combination of the culture-dependent and -independent techniques together with enzymatic assays revealed a substantial microbial diversity with several deteriogen microorganisms from the genera *Bacillus*, *Kocuria*, *Streptomyces* and *Geobacillus* and the fungal strains *Acrostalagmus luteoalbus*, *Bjerkandera adusta*, *Pleurotus pulmonarius* and *Trichothecium roseum*.

In the digital era it is very easy take a picture with a modern digital camera and it is equally easy to print it with ever newer and more rapid printing methods. But until the last decade of the 20^th^ century, it was not so simple, and photographs were printed on special photographic paper, composed of a light-sensitive emulsion containing of silver halide salts suspended in a colloidal material, usually gelatin, coated onto a paper, a resin coated paper or a polyester support^1^.

From the mid-19^th^ century to the beginning of the 20^th^ century, the most commonly used colloidal material was albumen. Albumen photographs were invented around 1850 by a French photographer, Louis Désiré Blanquart-Evrard. In these photographs, a paper sheet was covered by a layer of albumen, a type of protein found in egg whites, in order to bind the light-sensitive halide salts to the paper; they became the dominant form of photographic positives from 1855 to the turn of the 20^th^ century, peaking during the years 1860–90. The use of albumen paper greatly diminished around the year 1920^2^.

Many albumen-based photographs must exist in archives, galleries and museums around the world, both on display and in storage, and several of them are part of important historical collections; like the other historical objects in these collections, they too need to be safeguarded against aging and biodeterioration processes. It is already known how many microorganisms, mainly fungi, attack various types of archival materials, including books, parchments and photographic materials^3^,^4^, but, though many studies have been carried out to investigate the microflora responsible for the deterioration of different materials preserved in indoor environments^3^,^4^,^5^, few have examined the biodeterioration of photographic materials, and most of those that do have focused only on gelatin-based photographs^6^,^7^,^8^,^9^,^10^.

A methodological strategy for assessing the biodeterioration of albumen photographs, one of the most valuable “collections of memories” from the 19^th^ and 20^th^ centuries, is sorely lacking.

In this study we analyzed the material composition of and microflora present in two albumen photographs. Fourier-transform infrared spectroscopy (FTIR) and X-ray fluorescence (XRF), used to investigate the material, were combined with scanning electron microscopy (SEM), culture-dependent and culture-independent microbial analyses and different biodegradative agar assays in order to assess the nature of the microbial contaminants.

## Results

### Microscopic observation and chemical analysis

**Figure 1:**
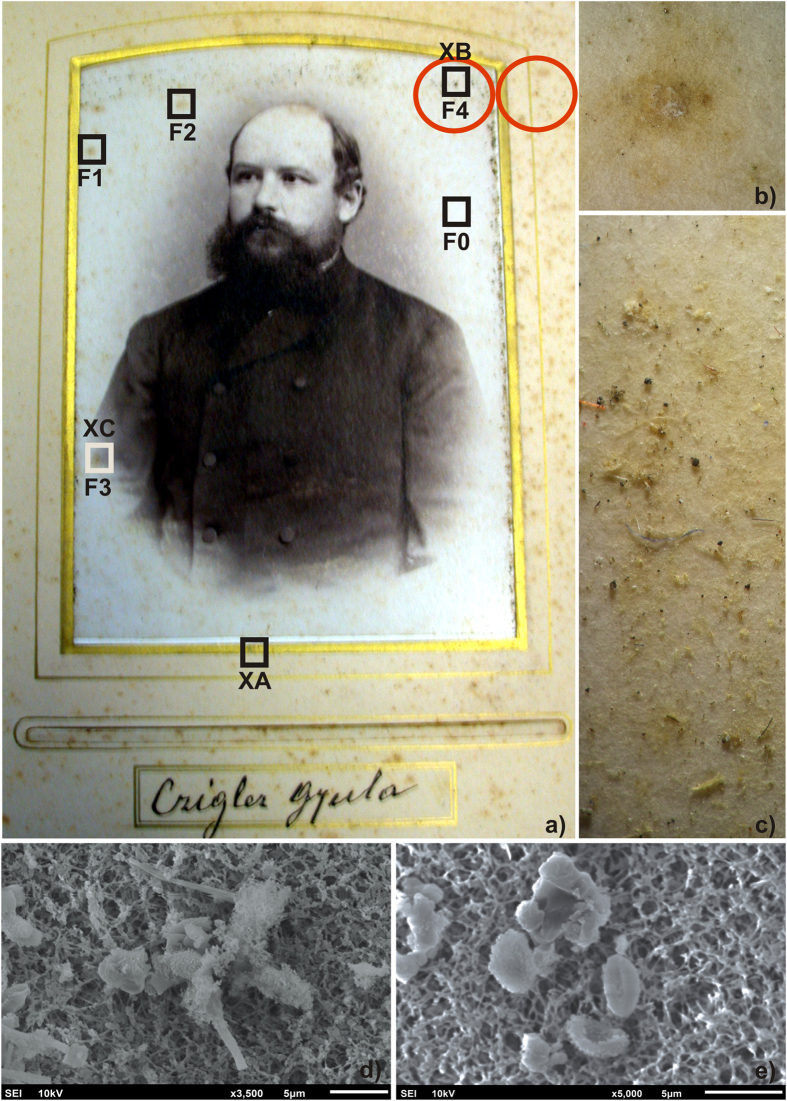
Gyula photograph. (**a**) The albumen surface is 9.7 × 13.9 cm; the size of the complete photograph including the cardboard is 10.8 × 16.2 cm. The microbial sampling sites are marked with two red circles. The sites F0–F4 were subjected to FTIR analysis, while the letters XA–XC indicate the sites where XRF measurements were performed. (**b**,**c**) Stereo microscope images with a magnification of 40× (site F4) and 20× (area near to site F4) respectively. (**d**,**e**) SEM photographs showing the fungal hyphae and spores recovered by a cellulose nitrate membrane. The photograph was supplied by the Slovak National Archive and is used with permission.

**Figure 2:**
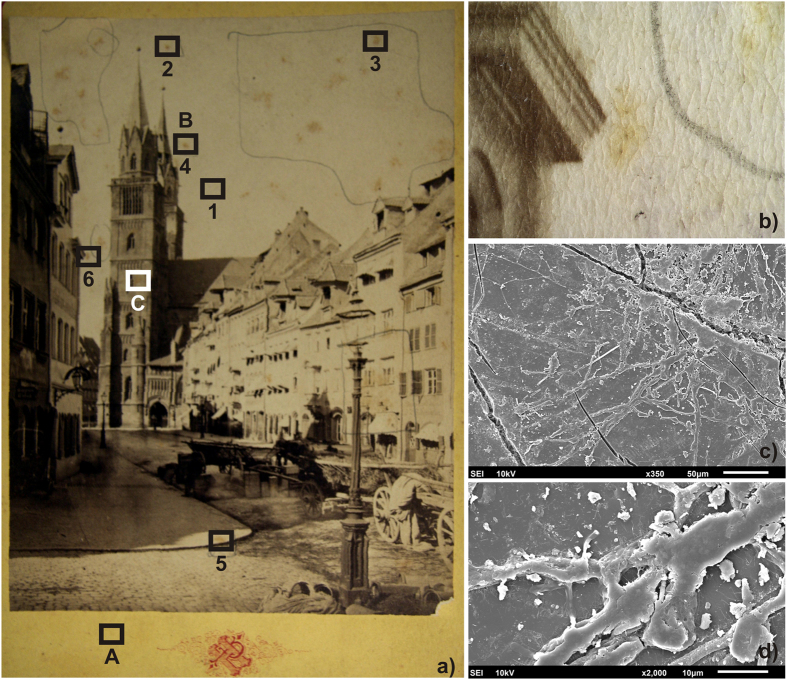
Antiquarian photograph. (**a**) The albumen surface measures 10.1 × 13.9 cm; the size of complete photograph including the cardboard is 10.9 × 16.6 cm. Numbers 1–6 indicate the sites where FTIR measurements were taken, while sites A–C were subjected to XRF examination. (**b**) A 20× stereo microscope image of site 6 (**c**,**d**) SEM photographs of site 2 where the presence of fungal hyphae and possible mineral structures is evident.

A preliminary observation of various parts of the two albumen prints was made using a stereo microscope. On the Gyula photograph (Fig. 1a) the presence of fungal contamination and different impurities on its surface near the sampling areas were evident (Fig. 1b,c). Cellulose nitrate membranes were used to lift microbial and (predominantly) fungal contaminants from the indicated areas of the photograph (red circles, Fig. 1a), and their presence was confirmed by the SEM analysis of a small portion of membrane (Fig. 1d,e). No microbial contamination could be clearly seen on the antiquarian print (Fig. 2a) using the stereo microscope (Fig. 2b), but SEM observation did reveal the presence of hyphae on the photograph’s surface (Fig. 2c,d).

**Figure 3:**
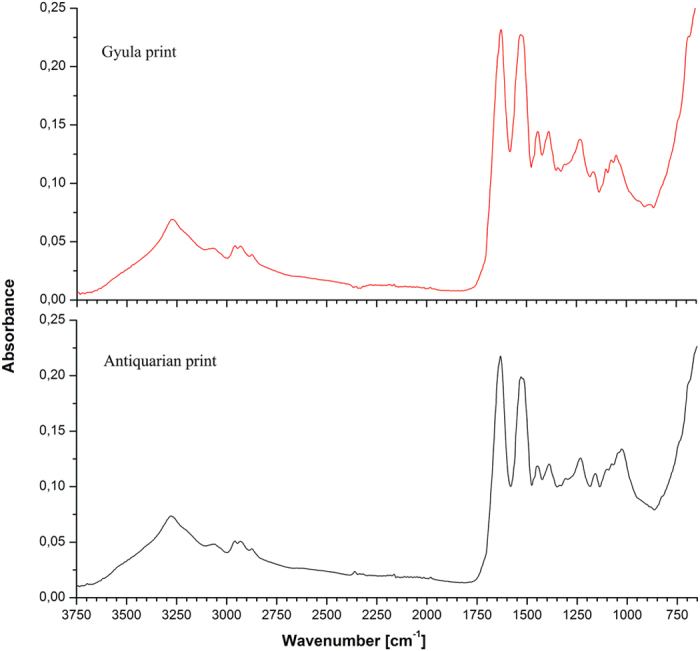
ATR-FTIR spectra of the two albumen prints, Gyula and antiquarian.

FTIR–ATR spectroscopy confirmed that the two photographs are, indeed, albumen prints. The typical absorption peaks for an albumen photograph appear between 1632 cm^−1^ and 1161 cm^−1^ and can be seen in Fig. 3.

**Figure 4:**
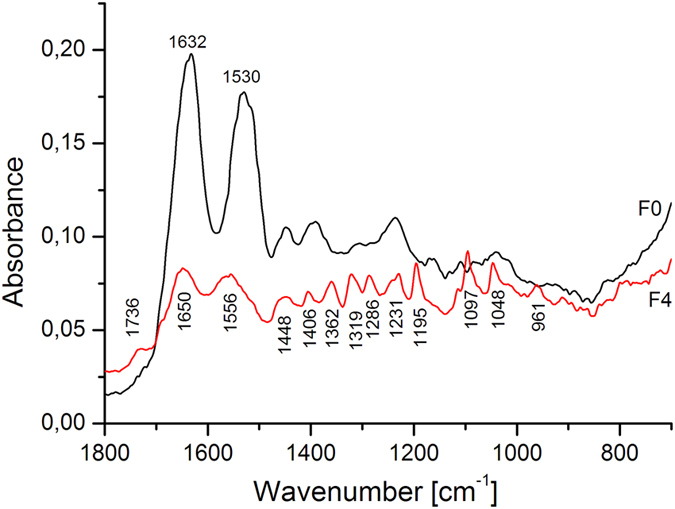
Comparison of μ-FTIR spectra between control site (F0) and the foxing site F4 of the Gyula print.

Figure 4 shows two different spectra from the Gyula print. The most intense peak in the F0 spectrum (the control sample) is at 1632 cm^−1^, corresponding to protein Amide I, the C = O and C-N stretching vibrations of (mainly) the peptide linkage. The peak at 1530 cm^−1^ belongs to Amide II, arising from the N-H bending and C-N stretching vibrations of the peptide bond Amide III is represented by the series of peaks in the 1400–1200 cm^−1^ 11 range. Because the albumin layers are deposited on paper supports in these kinds of photographs, the peaks in the range 1250–850 cm^−1^ indicate the presence of cellulose.

The μ-FTIR spectrum of the foxing stain (F4) showed shifts of the Amide I and Amide II bands to higher wavenumbers compared to F0 (Fig. 4). The foxing stain spectrum showed the greatest number of changes in the number of peaks and their shapes in the 1422–960 cm ^−1^ region, and a number of new peaks appeared (1736, 1406, 1362, 1319, 1286, 1231, 1195, 1097, 1048 and 961 cm^−1^). The functional groups corresponding to these peaks are described in Supplementary Table S1.

An XRF analysis of the Gyula photograph (Fig. 1a; XA–XC) detected high values of Ba and Ti in all three analyzed sites (Table 1), suggesting the presence of barium sulfate and titanium dioxide. The dark foxing site (Fig. 1a; XC) contained a small amount of silver which had been used to create the photographic image. Other elements detected in the two foxing stains included Ca, Fe and Sr. The cardboard of the antiquarian photograph (Fig. 2a; A) contained the elements Ag, Al, Fe, Cu, Mg and Zn (Table 1). Elevated concentrations of sulfur and silicon were also detected in all analyzed sites of this photograph. Bromine was present in both foxing sites (B and C), while the dark foxing site (C) also contained Ag and Al.

### Cultivation strategy and isolated microflora

Two different sampling procedures (direct inoculation and decimal dilution) and different medial compositions (some containing 3% NaCl) were used for the cultivation of microflora. Direct inoculation allowed only a few fungal strains to be isolated from the Gyula photograph (*Penicillium chrysogenum* P1_18_Fu, *Chaetomium elatum* P1_13_Fu and *Pleurotus pulmonarius* P1_15_Fu); the remaining fungal and bacterial strains were recovered by the inoculation of 10× diluted samples. Differences in the isolation power of the standard microbiological media used in this study (R2A and LB10; MEA and DRBC) were noted. In particular, only the two *Bacillus* strains (P1_1_5 and P1_2_8) isolated from Gyula photo grew on R2A and 59% of fungi were recovered on DRBC. MEA was only important for the isolation of the penicilli and aspergillli recovered (mainly) from the antiquarian photograph, but the sampling strategy for this photo also included the analysis of a piece of albumen print. It is possible to say therefore that the bacterial diversity of the albumen photograph samples was expressed better on LB10 and the fungal diversity on DRBC. The medium with 3% NaCl allowed different fungal strains to be isolated, only one of which, *Trichothecium roseum* (PF1_2_Fu), was also isolated on DRBC. The others, *Mucor plumbeus* (P1_Na_18_Fu), *Pleosporales* sp. (P1_Na_21_Fu), *Cephalosporium* sp. (PF1_Na_8_Fu) and *Cladosporium macrocarpum* (PF1_Na_30_Fu), were isolated only on salt medium, attesting to their slightly halophilic character. Only two halotolerant Actinobacteria were isolated, *Kocuria* sp. (PF1_Na_14) and *Streptomyces albidoflavus* (PF1_Na_10). All of the slightly halophilic microorganisms were isolated from the Gyula photograph samples (Table 2).

Only a few bacterial strains were isolated from the three samples (2 isolates from the Gyula photo, 7 from the album paper frame and 3 from the antiquarian photograph). Most species were members of the *Bacillus* genus, and they were isolated from the albumen substrate of both photographs. The bacterial microflora isolated from the paper frame contained Actinobacteria belonged to the genera *Kocuria* and *Streptomyces* (Table 2).

The fungal community was more diverse than its bacterial counterpart. Most isolated fungi belonged to the phylum Ascomycota; members of the phylum Basidiomycota, together with a single member of the subphylum Mucoromycotina, were isolated only from the albumen substrate of the Gyula photograph. The most common genus was *Penicillium*, and from the antiquarian photograph only penicillia and aspergilla were isolated. The fungal diversity of the album paper frame also included members of the genus *Cephalosporium*, *Trichothecium* and *Acrostalagmus* (Table 2).

### Culture-independent investigation

**Figure 5:**
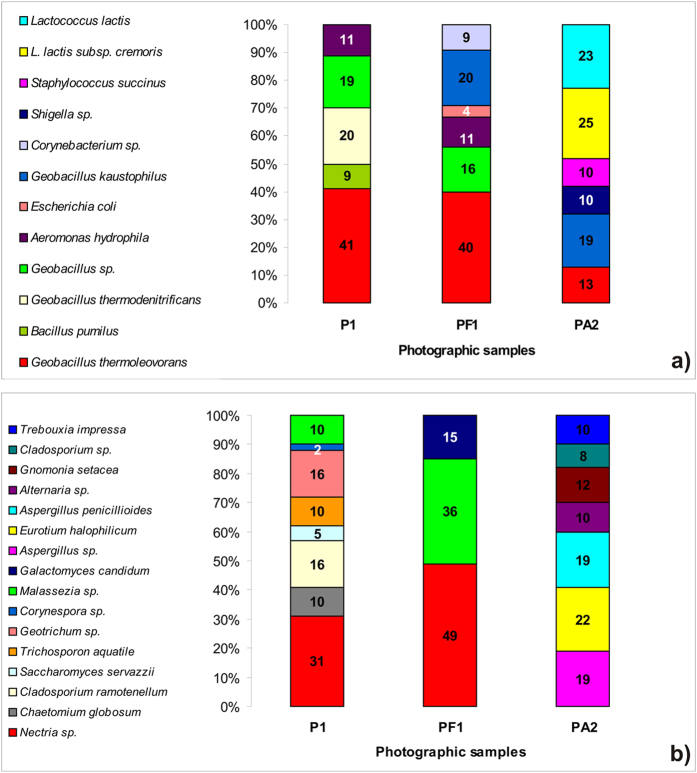
Microbial communities detected in photographic samples. Bacterial (**a**) and eukaryotic (**b**) diversity on photographic materials detected using the DGGE and clone library approach. P1: Gyula photo from Slovak National Archive; PF1: album paper frame of Gyula photo; PA2: antiquarian photograph.

Because not every microorganism in the microbial community is able to be isolated and cultivated, we also extracted and sequenced the communities’ total DNA to identify those organisms which resisted cultivation. The extracted bacterial DNA belonged mainly to Bacilli and Gammaproteobacteria; several *Corynebacterium* sequences were detected from the album paper frame sample, representing the only link with the Actinobacteria class. Specifically, the most common bacteria found in the samples from the Gyula photograph belonged to the genus *Geobacillus* which were 80% and 76%, respectively, of the population recovered from the albumen surface (P1) and the album paper frame (PF1). Some *Geobacillus* species were found only on the albumen surface (*G. thermodenitrificans*) while others were present only on the paper frame (*G. kaustophilus*). The third most common bacterium was *Aeromonas hydrophila*, found on both the albumen and the album, while *Bacillus pumilus* DNA was detected only on the albumen surface (Fig. 5a).

The bacterial community of the antiquarian print (PA2) was markedly different from the Gyula photograph: here *Lactococcus lactis* OTUs were the most abundant, reaching 48%, followed by *Geobacillus* OTUs (*G. kaustophilus* and *G. thermoleovorans*), with 32%, and *Staphylococcus succinus* and *Shigella* sp., 10% each (Fig. 5a; Supplementary Table S2).

Differences between the fungal communities of the Gyula photo and the album paper frame were noted: the DNA of *Nectria* and *Malassezia* members occurred in both samples, but *Galactomyces candidum* OTUs were detected only on the paper frame. The albumen material showed the greatest fungal divesrsity, with *Chaetomium*, *Cladosporium*, *Saccharomyces*, *Trichosporon*, *Geotrichum* and *Corynespora* OTUs all detected (Fig. 5b; Supplementary Table S2).

The photograph PA2 clone library showed yet another kind of fungal community, composed mainly of *Aspergillus* OTUs (38%) together with *Eurotium halophilicum* (22%), *Gnomonia setacea* (12%), *Alternaria* sp. (10%) and *Cladosporium* (8%). The DNA of the green algae *Trebouxia impressa* was also detected (Fig. 5b; Supplementary Table S2).

It should be noted that these cloning and sequencing approaches allowed the composition of the microbial communities colonizing the two photographs to be determined, but does not by itself indicate which of their members are responsible for the biodegradation.

### Biodegadative, catalase and peroxidase activities

**Figure 6:**
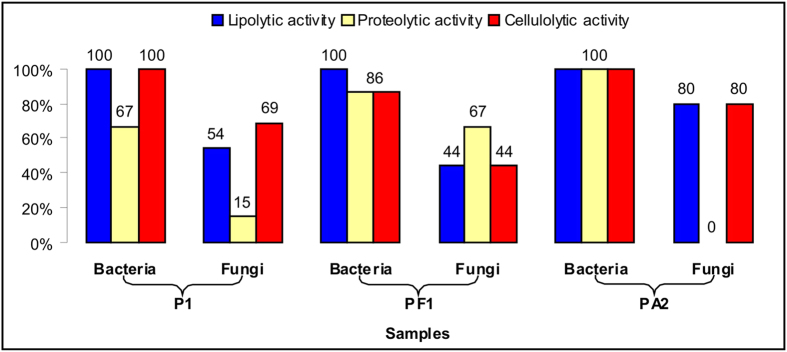
Percentage of hydrolytic abilities of isolated microflora. Distribution of hydrolytic activities of microorganisms isolated from different samples. P1: Gyula photo from Slovak National Archive; PF1: album paper frame of Gyula photo; PA2: photograph from antiquarian.

All isolated bacteria displayed significant biodegradation activities. The two *Bacillus* strains contaminating the Gyula photograph quickly produced a very intense halo around the colonies when cultivated on Spirit Blue (lipase activity) and Congo red (cellulase activity). The Actinobacteria from the album paper frame, members of the genus *Kocuria* and *Streptomyces*, exhibited strong lipolytic and proteolytic properties, (with the exception of *Kocuria rhizophila* PF1_1, which was positive only to the lipolytic assay). The three *Bacillus* sp. isolated from the antiquarian print, meanwhile, showed very similar biodegradative profiles, with weaker protelytic and lipolytic activities compared to those strains recovered from the other samples. The distribution of hydrolytic activities of the bacterial isolates is shown in Table 3 and Fig. 6. The *Bacillus* strains from both albumen photographs exhibited similar peroxidase activities (0.07–0.1 U/ml) and very high catalase activities, from the 1257 U/ml of PA2_1_3 to the 1340 U/ml of strain P1_1_5. The Actinobacteria from the album paper frame possessed weaker catalase activity than the *Bacillus* strains, with values between 436 U/ml for *Streptomyces* PF1_7 and 560 U/ml for *Kocuria rhizophila* PF1_1. The other *Kocuria* isolate (PF1_Na_14) exhibited 0.2 U/ml peroxidase activity, the highest from the bacteria (Table 3).

Nineteen fungal strains from a total of 27 isolates demonstrated at least one biodegradative property. The fungi isolated from the two albumen photographs showed feeble activities with respect to the bacteria. Figure 6 shows that only 15% of the Gyula print isolates possessed proteolytic activity, and that none of the fungi isolated from the antiquarian photograph did. The most active fungal isolates were P1_6_Fu *Bjerkandera adusta* (with marked lipolytic and cellulolytic activities), P1_15_Fu *Pleurotus pulmonarius* (characterized by both proteolytic and cellullytic abilities) and PA2_6_Fu *Aspergillus versicolor* (medium lipolytic and cellulolytic activities).

Sixty-seven percent of the fungi isolated from the album paper frame displayed proteolytic activity, but only 44% were able to produce lipases and cellulases (Fig. 6). The fungi *Trichothecium roseum* (PF1_ 2_Fu and PF1_Na_12_Fu) and *Acrostalagmus luteoalbus* (PF1_3_Fu and PF1_2_2_Fu) had the best hydrolytic properties, producing a positive reaction in all three biodegradative assays (Table 3).

The fungi showed stronger peroxidase activities than the bacteria, many of them equalling the best bacterial result (0.2 U/ml). The P1_15_Fu *Pleurotus pulmonarius* and P1_Na_21_Fu *Pleosporales* sp. isolates went higher, reaching 0.4 U/ml. The fungi with the highest catalase activities were isolated from the Gyula photograph: P1_6_Fu *Bjerkandera adusta*, P1_13_Fu *Chaetomium elatum*, P1_Na_18_Fu *Mucor plumbeus* and P1_Na_21_Fu *Pleosporales* sp. exhibited 350, 450, 600 and 500 U/ml, respectively (Table 3).

## Discussion

Albumen prints have important historical and cultural value, they represent the images of an epoch, the late 19^th^ and early 20^th^ centuries, when albumen was the most popular photographic binder. Albumen photographs are stored and exhibited in many galleries, museums, libraries and archives; it is therefore crucial to know their composition and the causes of their bio-deterioration.

The albumen paper prints were be prepared in different ways, but generally required the use of a very pure, metal-free paper support (metals may have caused black stains to appear in the final photographs). The albumen, derived from the clear white of fresh eggs, was mixed with NaCl or NH_4_Cl. The paper substrate was then coated by floating it in this salted albumin solution. After drying, the albumen paper print was sensitized by brushing it with a 10% AgNO_3_ solution, which readted with the salt to form the insoluble, light-sensitive AgCl. When this sensitized paper was exposed to sunlight, an image was formed by the reduction of the silver on surface of these AgCl crystals, which were embedded in the albumen layer. The albumen photograph was then washed in water, stabilized in a 15% sodium thiosulfate solution, and again washed^2^.

The chemical composition of the photographs examined here is not especially different from those analyzed in previous works^12^,^13^, and this investigation confirmed the utility of FTIR and XRF for noninvasively probing such sensitive materials.

The FTIR showed differences between the control sample (F0) and foxing areas (F1–F4) in the range 1736–960 cm^1^; these differences could arise from three sources: microbial contamination, impurities and degradation products of the albumen print. The interpretation of these results is difficult because the peaks from the albumen layer, paper support and biological contamination overlap. On the basis of the findings of Oberle-Kilic *et al.*^14^,^15^,^16^ it is reasonable to assume that the spectral differences arise from microbiological contaminations. Zotti *et al.*^17^ showed that the presence of active fungi on paper can be easily detected by FTIR analysis on the basis of the amide I and amide II bands around 1635 and 1540 cm^−1^ and a plateau between 1500 and 1200 cm^−1^. Our FTIR results showed that the albumen produces a FTIR spectrum very similar to that of microfilamentous fungi (Fig. 4, spectrum F0), making it impossible to clearly attest to the presence of fungi on albumen surface using FTIR alone^18^.

XRF analysis permitted the identification of the different inorganic elements from the various compounds used during the production of the albumen prints. Barium sulfate and titanium dioxide (detected on the Gyula photograph) were used for several purposes in photographic materials, including the creation of decorative elements on cardboard frames^2^. The presence of Si and Al, detected on the antiquarian photograph, suggests that kaolin [Si_2_Al_2_O_5_(OH)_4_] together with calcium carbonate (CaCO_3_) was used for coating the paper^19^. Bromine indicates that bromide salts (AgBr) were used for the production of the photograph. The other elements, such as sulfur and iron, were also detected in other photographic materials examined in a previous study^6^, and the increased amount of sulfur here may also indicate the presence of sulfur-containing degradation products from albumen or paper^20^. Cu, Mg and Zn are likely to be impurities. The absence of Ag in the pale foxing places (XB and B) indicates that it had been washed out during photographic processing. The lack of metals such as Au, Pt, and Se suggests that the two photographs were not toned^2^.

FTIR and XFR both showed that the albumen photographs are a mixture of proteins, cellulose and salts, which can be a suitable substrate for microbial colonization. Such biological contamination can sometimes be observed using a normal light microscope, as in the case of the Gyula photograph. Other studies have previously shown the advantages of using membranes for sampling cultural heritage objects^6^,^21^,^22^. The SEM analysis of a cellulose nitrate membrane performed here revealed fungal colonization, and can be considered a noninvasive alternative to SEM instruments with specialized sample chambers^6^.

Comparing the isolated microflora from these two albumen prints with those from similar items, for example gelatin-based photographs^6^,^7^,^8^,^9^,^10^, shows that there are some common members from the *Bacillus*, *Aspergillus* and *Penicillium* genera. Several fungi, such as *Pleurotus pulmonarius*, *Phlebia* sp., *Pleosporales sp.*, *Mucor plumbeus*, *Bjerkandera adusta*, *Chaetomium elatum*, had not previously been isolated on photographic materials, although some of them had been found on other items and in other museum and archival environments^23^,^24^,^25^,^26^. In particular, *Bjerkandera adusta* had already been isolated from a foxing stain on a 19th century book^23^.

There is a physical connection between the Gyula albumen print and album paper frame and the sampling membrane lifted microorganisms and spores colonizing both materials, but there were evident microbial differences between these two samples; the album paper frame had a diverse bacterial microflora, characterized by *Kocuria* and *Streptomyces* isolates, confirming the findings of previous studies related to cinematographic films, historical documents and archival atmospheres^4^,^5^,^7^,^9^.

To our knowledge, *Trichothecium roseum* and *Acrostalagmus luteoalbus* had not been previously found in this kind of habitat. The members of these two fungi displayed significant hydrolytic properties and catalase and peroxidase activities, indicating their ability to degrade composite substrates, but unfortunately no information about their enzymatic characteristics could be found in the literature. The hydrolytic and oxidoreductive abilities of other fungi, such as *Bjerkandera adusta*, *Pleurotus pulmonarius* and *Mucor plumbeus*, were confirmed by our investigation, and, in fact, the different biodegradation properties of these fungi have already found application^27^,^28^,^29^. Once again, the bacterial cohort exhibited dangerous lipolytic, proteolytic and cellulolytic activities against cultural heritage items^30^,^31^,^32^, though these activities could also be exploited for biotechnological applications^33^.

The hydrolytic properties of the isolates in this study were complemented by catalase and peroxidase activities. In this kind of dry envrionment, oxidoreductive enzymes may have a dual role. First, they may protect the bacterial cells against oxidative stress by removing the hydrogen peroxide produced as a byproduct of oxygen metabolism; in fact previous work showed an increase in catalase and peroxidase production in different kinds of microorganisms when cultivated in water-deficient conditions^34^,^35^,^36^. Second, it has recently been established that oxidoreductases are present during the biodegradation of cellulose materials by fungi^37^,^38^; indeed this recent finding allowed different oxidoreductive enzymes to be included in the carbohydrate-active enzymes database (CAZy)^39^. It is therefore possible to colclude that oxidoreductases are also involved in biodeterioration, and bio-degradation processes in support of the hydrolytic enzymes. Both roles suggest that the high catalase and peroxidase activities of the isolates could aid these microorganisms in better colonizing and utilizing the contaminated substrate.

Several previous studies^26^,^32^,^40^ have shown that a combination of culture-dependent and -independent methods provides better microbiological characterization than either method alone. Indeed, only the culture-independent approach was able to detect *Geobacillus* members in all analyzed samples. The presence of these thermophilic organisms was surprising because they normally require a growth temperature between 45–70 °C^41^. The most likely hypothesis is that the *Geobacillus* strains contaminated the albumen supports during their preparation. Different methods were used to harden the albumen layers, including steaming and storing the albumen-coated papers inside a warm hayloft for half a year^42^. The temperature of a hayloft can sometimes reach 50 °C or more^43^, providing an optimum environment for the growth of *Geobacillus* strains. These bacteria were also detected on the paper frame of the photo album which was in contact with the Gyula print. If *Geobacillus* contamination was intrinsic in the Gyula print, the album paper frame could have become contaminated when the photo album was stored in conditions allowing the growth of these microorganisms. *Geobacillus* members were also detected in the albumen layer of the antiquarian photograph, making it seem likely that *Geobacillus* tended to colonize these types of photographs during their manufacturing. These microorganisms can cause serious deterioration problems due to to their known hydrolytic properties^41^.

Lactococci were the most prevalent group on the antiquarian print, and their presence too may be connected with the hardening of the albumen layer of this photo, perhaps inside a hayloft where lactic acid bacteria are known to participate in silage fermentation^43^.

The fungal microflorae detected in the three samples by the DGGE-cloning approach are complementary to those isolated by cultivation. Only a few links were found, namely the *Aspergillus* and *Chaetomium* strains, which were detected by both microbial investigation methods in, respectively, the antiquarian print and the Gyula print.

The paper frame of the photo album showed the lowest fungal diversity; the same fungal members (*Nectria* sp. and *Malassezia* sp.) were detected in both the album and the Gyula print, although the fungal community of the albumen print was richer, containing many other species, several of which, *Trichosporon*, *Geotrichum*/*Galactomyces*, *Chaetomium* and *Cladosporium*, have already been found in photographic materials or archival documents^6^,^10^,^44^,^45^. Fungal species of the genera *Nectria*, *Corynespora* and *Saccharomyces* were also seen, which have not frequently been encountered in archival environments. Different members of *Nectria* and *Corynespora* are plant pathogens, so they possess significant lignocellulolytic abilities^46^,^47^. *Malassezia* strains are normally isolated from epidermises or skin scalp^48^, so their presence on these samples probably is likely due to handling of the photo-book over time.

Three particular eukaryotic organisms were only detected in the antiquarian albumen photograph: *Eurotium halophilicum,* a xerophilic species previously found in archival items^21^,^49^, *Trebouxia impressa* and *Gnomonia setacea*. Algae of the *Trebouxia* genus are generally photobiont lichens and it is not surprising to find them on surfaces exposed to other kinds of environments^32^,^50^; they were very recently identified on da Vinci’s self-portrait^21^. *Trebouxia* species are heavy-metal tolerant, and their presence here may be due to the traces of different metals found in the photograph (this property has also lead to their use in detoxification applications^51^,^52^). *Gnomonia setacea* is an endophytic species frequently isolated from *Betula* trees^53^, but little is currently known about their hydrolytic potential; to our knowledge members of this species have never before been detected in archival objects and environments.

## Conclusion

Only few studies have analyzed albumen photographs and, to our knowledge, none of them treated the biodeterioration of this important part of our heritage. Safeguarding our cultural heritage requires methods able to identify the construction materials of historical objects and to diagnose the potential biodeteriogens in order to develop targeted conservation and restoration strategies. This study has illustrated a possible armamentarium of techniques and investigative approaches for the analysis of albumen prints.

FTIR and XRF were useful in analyzing the photographic materials and the composition of foxing stains. The use of cellulose nitrate membranes for sampling the microflora on the surface of the photographs and their subsequent observation by SEM provides an easy, noninvasive procedure for acquiring a preliminary idea of the extent of microbial contamination. Exploiting different kinds of microbiological media allowed a diverse microbial community (especially the fungal) to be isolated. The cloning and sequencing approach together with DGGE screening of the clones revealed different species from those present in the cultivation trial. This combination of culture-dependent and culture-independent methods revealed complex microbial communities in all analyzed samples. Finally, enzyme assays allowed the most deteriogenic microorganisms to be identified.

## Methods

### Samples

Two albumen photographs were the subject of this investigation. The first photograph (Gyula; P1) is dated 1889; it was produced by an important photographer, Max Stern (1836–1901), and is one of 218 photographs contained in the official photo album of the district of Trenčin. It has been stored in the Slovak National Archives since 1991. The Gyula photograph (Fig. 1) is surrounded by the paper frame of the photographic album (Gyula; PF1) and it has clearly been affected by an easily visible microbial contamination; several different foxing stains can be seen on its surface. The Gyula photograph was offered for investigation, before various conservation treatments were undertaken, in order to determine potential microbial contaminants. Only noninvasive methods were applied to this photo.

The second albumen photograph was bought by a local antiquarian and brought to the laboratory inside a sterile bag. On this antiquarian photo (PA2) (Fig. 2) only foxing stains were visible to the naked eye. This photograph “was sacrificed”, using invasive investigation methods, in order to better understand the microbial contamination of albumen photos and to compare it with the Gyula photograph.

### Microscopic observation

Both photographs were examined with a stereo microscope (Olympus SZX9; Tokyo, Japan) at 20–40× magnification and light incident at a 45° angle.

A portion of the 25 mm cellulose nitrate membrane used for the microbiological sampling of the Gyula photograph surface (F4, area outlined by red circle, Fig. 1a) and a foxing stain (point 2 on Fig. 2a) cut from the antiquarian photograph were examined by SEM (Jeol JSM 6610, Tokyo, Japan) using the facilities offered by the Institute of Materials and Machine Mechanics of the Slovak Academy of Sciences. Prior to SEM observation, the samples were sputtered with gold ions.

### μ-FTIR and ATR-FTIR spectroscopy

μ-FT-IR spectra were measured using the attenuated total reflection technique using a μ-FTIR-ATR Varian 610-IR (Agilent Technology, Santa Clara, USA), equipped with a Ge/KBr beamsplitter and extended with a Continuum Microscope and a liquid nitrogen cooled MCT detector. Measurements were performed at a frequency of 20 kHz in the 4000–600 cm^−1^ range at a resolution of 4 cm^−1^, averaging 400 acquisitions per sample. The spectra were processed by the Varian Resolution Pro program (Agilent Technology). Four places in the Gyula print with foxing stains (F1–F4; Fig. 1a) were analyzed by μ-FTIR. The place F0, which lacked apparent damage, was treated as the control in order to determine the photographic technique. A similar strategy was applied to the antiquarian photograph where points 2–6 evidenced foxing alterations and point 1 (Fig. 2a) was used to identify the photographic technique.

The reflectance spectra of albumen prints in the infrared region were also measured on an Excalibur FTS 3000MX (Digilab, Randolph, USA) spectrometer with an ATR adapter containing a diamond crystal. Measurements were taken at a frequency of 20 kHz in the 4000–600 cm^−1^ range at a resolution of 4 cm^−1^, averaging 60 acquisitions per sample. The ATR spectra were processed by the Varian Resolution Pro (Agilent Technology) program.

### XRF measurement

XRF measurements were performed at three different points (the cardboard around the photographs and foxing stains from pale and dark sites; Fig. 1a XA-XC; Fig. 2a A–C). A portable XRF spectrometer was used for both photographs (X-MET5100; Oxford Instruments, Abingdon, UK). The X-ray tube was operated at a voltage of 45 kV and a current of 40 mA, the scanning time was 60 s and the spectral resolution was 150 eV.

### Sampling and microflora isolation

A sterile, 25 mm cellulose nitrate membrane (Sartorius, Goettingen, Germany) was pressed onto a contaminated section of the albumen part of the Gyula photograph, and a second membrane was pressed onto the album paper frame (Fig. 1a red circles).

The sampling of the antiquarian albumen print (PA2) was performed by cutting off a piece of the photograph (Fig. 2a, point 3). This piece of albumen print was suspended in 2 ml of physiological solution; after vortexing, 200 μl of the resultant suspension were plated in the same type of agar media as in the membrane approach described below. The remainder of the suspension, including the photograph fragments, was used for DNA extraction.

Each membrane was cut in five pieces: one piece was used for SEM observation (see above), 3 pieces were used for cultivation and one piece was used for DNA extraction (see below).

Cultivation was done in two different ways: (i) in direct inoculation, two pieces of membrane from each sample were put on 10× diluted Luria Bertani (LB10) agar plates for bacteria and on Malt Extract Agar (MEA) for fungi; (ii) in the membrane approach, a different piece of membrane for each sample was suspended in physiological solution, diluted 10× and plated onto agar dishes specific for the growth of bacteria or fungi. For bacterial isolation, the membrane approach used R2A (Oxoid, Basingstoke, UK), LB10 and gelatin agar with 3% NaCl (R2A-Gel-NaCl); for fungal isolation, the method used Dichloran Rose Bengal Chloramphenicol (DRBC; Hi-Media, Bombay, India), MEA (Hi-Media) and R2A-Gel-NaCl. The gelatin medium was prepared using R2A agar (Oxoid, Basingstoke, UK) plus 3% NaCl. This medium was autoclaved separately and then 0.4% of sterilized gelatin was added (Sigma-Aldrich, Germany) along with the amount of 20% MgSO_4_ (autoclaved separately) needed to reach a final MgSO_4_ concentration of 2%. All bacterial and fungal plates were incubated at room temperature (22–26 °C) for five days to 2 weeks. The agar media were supplemented with either actidione (50 mg l^−1^; Fluka, Seelze, Germany) or chloramphenicol (50 mg l^−1^; Sigma–Aldrich, Seelze, Germany) in order to avoid the growth of fungi and bacteria respectively.

### DNA extraction from isolated microflora and identification

Microorganisms were selected on the basis of differing morphology and transferred to new agar plates to obtain pure colonies. The purified fungal isolates were maintained on Sabouraud (SAB) slants, the bacteria on plates of Tryptone-Soya Agar (TSA; Oxoid). For DNA extraction from fungal strains, the isolates were inoculated in SAB broth at 28 °C until growth; they were then separated from the broth by filtration through sterile filter paper, followed by DNA extraction with Ron’s fungal DNA mini kit (Bioron, Ludwigshafen, Germany), according to the instructions of the manufacturer. The ITS region of rDNA was amplified with the primers ITS1 (5′-TCC GTA GGT GAA CCT GCG G-3′) and ITS4 (5′-TCC TCC GCT TAT TGA TAT GC-3′)^54^. The 25 μl PCR mixture contained 50 pmol of each primer, 200 μmol l^−1^ of dNTP (Life Technologies, Gaithersburg, Maryland, USA), 1.5 U HotStar Taq plus DNA polymerase (Qiagen, Hilden, Germany), 1× PCR buffer and 3 μl of the extracted DNA (the PCR template). The PCR program consisted of an initial denaturation at 94 °C for 5 min, followed by 30 cycles (denaturation at 94 °C for 30 s, annealing at 54 °C for 45 s, extension at 72 °C for 1 min) and a final polymerization step at 72 °C for 10 min.

For DNA extraction from bacteria, fresh bacterial colonies were collected from the TSA plates and their DNA was isolated using InstaGene Matrix (Biorad, Hercules, CA, USA) following the instructions of the manufacturer; this extracted DNA was then used as a PCR template. The bacterial isolates were identified by partial sequencing of the 16S rDNA using PCR with the primers 27f (5′-AGA GTT TGA TCC TGG CTC AG-3′) and 685r (5′-TCT ACG CAT TTC ACC GCT AC-3′)^55^; the PCR conditions and program were the same as for the ITS procedure.

The resulting PCR products from both fungal and bacterial strains were purified using ExoSAP-IT (Affymetrix, Cleveland, Ohio, USA) and sequenced at a commercial facility (GATC-Biotech, Konstanz, Germany). The resulting sequences were directly compared with those in GenBank using blast program (http://blast.ncbi.nlm.nih.gov/Blast.cgi) and were subsequently deposited in GenBank under the accession numbers KT200239–KT200250 (bacterial isolates) and KT200251–KT200277 (fungal isolates).

### Total DNA extraction and 16S rRNA, 28S rRNA gene amplification

The total microbial DNA was extracted by chaotropic solid-phase extraction (DNeasy Blood & Tissue Kit; Qiagen) from the remaining cellulose nitrate membranes (from the Gyula photo and its surroundeding paper frame) and from the suspension containing the antiquarian photograph fragments (point 3 on Fig. 2a) by the protocol described by Bučková *et al.*^6^

The bacterial 16S rDNA and eukaryotic 28S rDNA fragments were amplified in two steps. The first step involved primers 27f and 685r oriented towards the bacterial 16S rRNA gene. For the amplification of the eukaryotic 28S rRNA gene, the universal primers NL1 (5′-GCA TAT CAA TAA GCG GAG GAA AAG-3′) and NL4 (5′-GGT CCG TGT TTC AAG ACG G-3′)^56^ were used. The 25 μl PCR mixtures contained 50 pmol of each primer, 2.5 mmol l^−1 ^MgCl_2_, 200 μmol l^−1^ of dNTP, 1.5 U HotStar Taq plus DNA polymerase (Qiagen), 1 × PCR buffer and 3 μl (4.2–8.45 ng μl^−1^) of extracted DNA. The PCR was performed with an initial 5 min denaturation step at 95 °C followed by 35 cycles of denaturation at 94 °C for 30 s, annealing at 54 °C (amplification of 16S rRNA gene) and at 56 °C (amplification of 28S rRNA gene) for 1 min, and extension at 72 °C for 1 min, subsequently followed by a 10 min extension step at 72 °C. For each DNA target, four reactions of 25 μl (100 μl altogether) were produced. The four reactions of each DNA target were mixed together and the specificity of amplification was checked by agarose gel electrophoresis. A part of these PCR products were set aside for the construction of clone libraries (see below).

### Denaturing gradient gel electrophoresis fingerprint analysis

The remainder of the PCR product from the first step (1 μl) was used as a template in the second amplification step, a semi-nested PCR for each DNA target. The 16S rRNA gene was re-amplified with primers 518f (5′-CCA GCA GCC GCG GTA AT-3′) and 685r-GC (5′-CGC CCG CCG CGC GCG GCG GGC GGG GCG GGG GCA CGG GGG GTC TAC GCA TTT CAC CGC TAC-3′); the primers NL1f-GC (5′- CGC CCG CCG CGC GCG GCG GGC GGG GCG GGG GCA CGG GGG GGC ATA TCA ATA AGC GGA GGA AAA G-3′) and LS2 (5′- ATT CCC AAA CAA CTC GAC TC-3′) were used for the semi-nested amplification of the 28S rRNA gene. The PCR conditions were the same as above. Denaturing gradient gel electrophoresis (DGGE) for bacterial and eukaryotic amplicons was performed as described by Pangallo *et al.*^57^ to produce DGGE fingerprinting profiles of the bacterial and eukaryotic communities.

### Construction of clone libraries and screening of clones

Clone libraries were produced from a portion of the PCR products from the first PCR amplification step following a previously described protocol^57^. Briefly, the PCR products were purified with the QIAquick PCR purification kit (Qiagen), ligated into a pGEM-T Easy vector (Promega,Madison, Wisconsin, USA), transformed into *E. coli* XLI-Blue, and spread on Luria Bertani plates with 100 μg ml^−1^ ampicillin 0.1 mM, X-Gal and 0.2 mM IPTG. To confirm the presence of the desired inserts, 100 white colonies of each clone library were picked and directly PCR screened for the presence of the inserts using the vector specific primers SP6 and T7^57^. Positive clones from each library were analyzed using the by semi-nested PCR DGGE procedure described above. The profiles of individual clones were compared with each other and with that of the whole community. A diagram schematically outlining the complete cloning and DGGE screening strategy is shown in Supplementary Figure S1. The PCR products of clones with different profiles were sequenced in order to identify the microorganisms colonizing the albumen prints. To sequence these products, they were first purified using ExoSAP-IT (Affymetrix, Cleveland, USA) and sequenced using the SP6 primer by a commercial facility (GATC Biotech, Konstanz, Germany). For microorganism identification, the sequences were compared directly with those in GenBank using blast program (http://blast.ncbi.nlm.nih.gov/Blast.cgi). These sequences were deposited in GenBank under accession numbers KT200278–KT200296 (bacterial clones) and KT200297–KT200316 (eukaryotic clones).

### Hydrolytic assays, catalase and peroxidase activities

Several agar plate tests were applied to assess the proteolytic (R2A-Gel agar), cellulolytic (Congo red agar) and lypolitic (Spirit blue agar) abilities of isolated strains as described by Šimonovičová *et al.*^58^. R2A-Gel agar plates were prepared by mixing autoclaved R2A Agar (Oxoid) with 0.4% of sterilized gelatin (Sigma–Aldrich, St. Louis, USA). The Congo-Red agar contained 0.5 g KH_2_PO_4_, 0.25 g MgSO_4_, 2 g cellulose, 0.2 g Congo-Red, 2 g gelatin, and 15 g agar in 1 l distilled water at pH 6.8–7.2. Spirit Blue agar was prepared by suspending 32.15 g of Spirit Blue agar (Himedia) in 1000 ml distilled water, autoclaved, cooled down to 50 °C and supplemented with 30 ml of lipase substrate (1 ml of Tween 80, 400 ml of warm distilled water and 100 ml of olive oil; sterilized by autoclaving). This was slow mixed and poured into Petri dishes. All chemicals, if not otherwise specified, were bought from Sigma–Aldrich. All assays were performed in triplicate using 60 mm plates incubated at room temperature (22–26 °C) generally for 3–7 days.

Catalase (CAT; EC 1.11.1.6) activity was determined spectrophotometrically at pH 7.0 by monitoring the decomposition of H_2_O_2_ at 240 nm with an extinction coefficient of 43.6 M^−1 ^cm^−1^. One unit (1 U) of catalase activity was defined as the amount of enzyme that catalyzes the decomposition of 1 μmol of H_2_O_2 _per min^59^.

Peroxidase (POX; E.C.1.11.1.7) activity was assayed spectrophotometrically at pH 5.5 by monitoring the oxidation of *o*-dianisidine dihydrochloride at 460 nm (ε460 nm = 11.3 × 103 M^−1^). One unit of peroxide activity (U) was defined as the amount of activity that produces 1 μmol of oxidized *o*-dianisidine per minute^60^.

## Additional Information

**How to cite this article**: Puškárová, A. *et al.* Microbial communities affecting albumen photography heritage: a methodological survey. *Sci. Rep.* **6**, 20810; doi: 10.1038/srep20810 (2016).

## Supplementary Material

Supplementary Information

## Figures and Tables

**Table 1:** XRF analysis of the Gyula and antiquarian photographs performed at three different sites: the cardboard frame (XA; A), pale foxing sites (XB; B) and dark foxing sites (XC; C).

Elements	Gyula photograph			Antiquarian photograph		
	Cardboard frame (XA) %	Foxing PS (XB) %	Foxing DS (XC) %	Cardboard frame (A) %	Foxing PS (B) %	Foxing DS (C) %
Ag	n.d.	n.d.	0.1 ± 0.0	0.5 ± 0.1	n.d.	1.1 ± 0.2
Al	n.d.	n.d.	n.d.	0.9 ± 0.1	n.d.	0.2 ± 0.0
Ba	9.76 ± 0.1	8.81 ± 0.1	11.83 ± 0.1	n.d.	n.d.	n.d.
Br	n.d.	n.d.	n.d.	n.d.	0.3 ± 0.0	0.4 ± 0.1
Ca	0.2 ± 0.0	0.3 ± 0.1	0.4 ± 0.0	0.4 ± 0.1	0.3 ± 0.1	0.6 ± 0.0
Cu	n.d.	n.d.	n.d.	0.5 ± 0.0	n.d.	n.d.
Fe	0.1 ± 0.0	0.1 ± 0.0	0.2 ± 0.0	0.3 ± 0.0	0.2 ± 0.0	0.4 ± 0.0
Mg	n.d.	n.d.	n.d.	0.9 ± 0.1	n.d.	n.d.
S	n.d.	n.d.	n.d.	2.5 ± 0.2	2.8 ± 0.1	3.2 ± 0.1
Si	n.d.	n.d.	n.d.	2.5 ± 0.1	0.8 ± 0.0	0.6 ± 0.0
Sr	0.6 ± 0.0	0.5 ± 0.0	0.6 ± 0.0	n.d.	n.d.	n.d.
Ti	11.4 ± 0.1	5.9 ± 0.0	6.4 ± 0.04	n.d.	n.d.	n.d.
Zn	n.d.	n.d.	n.d.	n.d.	0.3 ± 0.0	n.d.

n.d.: not detected.

**Table 2:** Bacterial and fungal strains isolated from albumen photographs and album paper frame.

Sample of isolation	Strain	Medium of isolation	Microorganisms identified based on the highest 16S rDNA and ITS similarity scores	Accession Number
Gyula albumen photograph	P1_2_8	R2A	KF844070 *Bacillus simplex* 561/561 (100%)	KT200239
	P1_1_5	R2A	KF995632 *Bacillus* sp. 613/614 (99%)	KT200240
	P1_1_Fu	DRBC	GU731546 *Bjerkandera adusta* 551/551 (100%)	KT200251
	P1_2_Fu	DRBC	KJ831970 *Phlebia* sp. 639/654 (98%)	KT200252
	P1_3_Fu	MEA	KJ583240 *Penicillium* sp. 475/477 (98%)	KT200253
	P1_4_Fu	MEA	KJ653464 *Penicillium* sp. 501/501 (100%)	KT200254
	P1_6_Fu	DRBC	KP233879 *Bjerkandera adusta* 451/451 (100%)	KT200255
	P1_13_Fu	DRBC	KC109758 *Chaetomium elatum* 511/511 (100%)	KT200256
	P1_2_3_Fu	DRBC	KJ831970 *Phlebia* sp. 639/655 (98%)	KT200257
	P1_1_20_Fu	DRBC	KC109758 *Chaetomium elatum* 511/512 (99%)	KT200258
	P1_10_Fu	DRBC	KJ653464 *Penicillium* sp. 520/521 (99%)	KT200259
	P1_15_Fu	DRBC	KM985673 *Pleurotus pulmonarius* 399/401 (99%)	KT200260
	P1_Na_18_Fu	R2A-Gel-NaCl	JX537955 *Mucor plumbeus* 401/401 (100%)	KT200261
	P1_Na_21_Fu	R2A-Gel-NaCl	HQ207056 *Pleosporales* sp. 481/496 (97%)	KT200262
	P1_18_Fu	DRBC	JX139710 *Penicillium chrysogenum* 530/530 (100%)	KT200263
Gyula album paper frame	PF1_1	LB10	KC429605 *Kocuria rhizophila* 620/621 (99%)	KT200241
	PF1_2	LB10	KJ139433 *Streptomyces* sp. 611/611 (100%)	KT200242
	PF1_5	LB10	LN774467 *Staphylococcus epidermidis* 639/641 (99%)	KT200243
	PF1_7	LB10	KP128847 *Streptomyces* sp. 608/609 (99%)	KT200244
	PF1_30	LB10	KP128847 *Streptomyces* sp. 622/624 (99%)	KT200245
	PF1_Na_14	R2A-Gel-NaCl	KM874399 *Kocuria* sp. 619/621 (99%)	KT200246
	PF1_Na_10	R2A-Gel-NaCl	KP122209 *Streptomyces albidoflavus* 505/509 (99%)	KT200247
	PF1_Na_8_Fu	R2A-Gel-NaCl	HQ630979 *Cephalosporium* sp. 517/526 (98%)	KT200264
	PF1_2_Fu	DRBC	KJ466977 *Trichothecium roseum* 371/375 (99%)	KT200265
	PF1_3_Fu	DRBC	KM853014 *Acrostalagmus luteoalbus* 311/311 (100%)	KT200266
	PF1_2_2_Fu	DRBC	KM853014 *Acrostalagmus luteoalbus* 410/411 (99%)	KT200267
	PF1_4_Fu	MEA	KJ653464 *Penicillium* sp. 521/522 (99%)	KT200268
	PF1_6_Fu	MEA	KJ653464 *Penicillium* sp. 471/471 (100%)	KT200269
	PF1_5_Fu	MEA	KJ653464 *Penicillium* sp. 521/521 (100%)	KT200270
	PF1_Na_12_Fu	R2A-Gel-NaCl	EF589898 *Trichothecium roseum* 528/541 (98%)	KT200271
	PF1_Na_30_Fu	R2A-Gel-NaCl	KM396371 *Cladosporium macrocarpum* 497/499 (99%)	KT200272
Antiquarian photograph	PA2_1_1	LB10	KP178217 *Bacillus subtilis* 631/631 (100%)	KT200248
	PA2_1_2	LB10	KP178217 *Bacillus subtilis* 630/631 (99%)	KT200249
	PA2_1_3	LB10	KP178217 *Bacillus subtilis* 636/636 (100%)	KT200250
	PA2_4_Fu	DRBC	JN368454 *Penicillium chrysogenum* 533/534 (99%)	KT200273
	PA2_6_Fu	MEA	JX156356 *Aspergillus versicolor* 513/514 (99%)	KT200274
	PA2_3_Fu	MEA	LN809060 *Aspergillus versicolor* 501/502 (99%)	KT200275
	PA2_5_Fu	MEA	JN624909 *Penicillium thomii* 517/522 (99%)	KT200276
	PA2_2_Fu	MEA	EU910586 *Penicillium thomii* 525/526 (99%)	KT200277

MEA: Malt Extract Agar; DRBC: Dichloran Rose Bengal Chloramphenicol; LB10: 10x diluted Luria Bertani; R2A-Gel-NaCl: R2A supplemented with gelatin and 3% of NaCl.

**Table 3:** Hydrolytic abilities and catalase and peroxidase activities of microorganisms isolated from different samples.

Sample of isolation	Isolates	Lipolytic activity Sprit Blue Agar	Proteolytic activity Gelatin Agar	Cellulolytic activity Congo Red	Catalase activity U/ml	Peroxidase activity U/ml
Gyula albumen photograph	P1_2_8 *Bacillus simplex*	+++	++	+++	1270	0.07
	P1_1_5 *Bacillus* sp.	+++	++	+++	1340	0.08
	P1_1_Fu *Bjerkandera adusta*	+++	–	+++	343	0.2
	P1_ 2_Fu *Phlebia* sp.	+++	–	++	240	0.2
	P1_2_3_Fu *Phlebia* sp.	+++	–	++	237	0.2
	P1_4_Fu *Penicillium* sp.	–	–	+	270	–
	P1_6_Fu *Bjerkandera adusta*	+++	–	+++	350	0.2
	P1_13_Fu *Chaetomium elatum*	+	+	++	450	0.15
	P1_15_Fu *Pleurotus pulmonarius*	–	+++	++	200	0.4
	P1_Na_18_Fu *Mucor plumbeus*	++	–	++	600	0.2
	P1_Na_21_Fu *Pleosporales* sp.	++	–	++	500	0.4
Gyula album paper frame	PF1_1 *Kocuria rhizophila*	+++	–	++	560	0.1
	PF1_2 *Streptomyces* sp.	+++	+++	++	550	0.07
	PF1_5 *Staphylococcus epidermidis*	+++	+++	–	92	–
	PF1_7 *Streptomyces* sp.	+++	+++	++	436	0.09
	PF1_30 *Streptomyces* sp.	+++	+++	++	441	0.09
	PF1_Na_10 *Streptomyces albidoflavus*	+++	+++	++	471	0.1
	PF1_Na_14 *Kocuria* sp.	+++	+++	++	440	0.2
	PF1_ 2_Fu *Trichothecium roseum*	+++	+++	++	220	0.2
	PF1_3_Fu *Acrostalagmus luteoalbus*	+++	+++	++	257	0.2
	PF1_2_2_Fu *Acrostalagmus luteoalbus*	+++	+++	++	250	0.2
	PF1_6_Fu *Penicillium* sp.	–	+	–	350	0.12
	PF1_Na_8_Fu *Cephalosporium* sp.	–	+	–	340	0.2
	PF1_Na_12_Fu *Trichothecium roseum*	+++	+++	++	220	0.2
Antiquarian photograph	PA2_1_1 *Bacillus subtilis*	++	++	+	1260	0.1
	PA2_1_2 *Bacillus subtilis*	++	++	+	1262	0.1
	PA2_1_3 *Bacillus subtilis*	++	++	+	1257	0.1
	PA2_6_Fu *Aspergillus versicolor*	++	–	++	290	0.1
	PA2_3_Fu *Aspergillus versicolor*	+	–	++	300	0.05
	PA2_5_Fu *Penicillium thomii*	+	–	++	252	0.1
	PA2_2_Fu *Penicillium thomii*	+	–	++	255	0.1

+++: quick and extensive positive reaction; ++: strong positive reaction; +: weak p 838 ositive reaction; -: no reaction.
