# Supplementary material for: Microbial communities affecting albumen photography heritage: a methodological survey
Source: Sci Rep. 2016 Feb 11;6:20810. doi: 10.1038/srep20810 (PMC4749957; doi:10.1038/srep20810)
Supplement: Supplementary Information [file srep20810-s1.pdf]

Supplementary Information

Microbial communities affecting albumen photography heritage: a methodological survey

Andrea Puškárová[1], Mária Bučková[1], Božena Habalová[2], Lucia Kraková[1], Alena Maková[2], Domenico Pangallo[1, 3, *]

[1] Institute of Molecular Biology, Slovak Academy of Sciences, Dúbravská cesta 21, 84551 Bratislava, Slovakia

[2] Slovak National Archives, Department of Archival Preservation, Drotárska cesta 42, 84005 Bratislava, Slovakia

[3] Caravella, Ltd., Tupolevova 2, 85101 Bratislava, Slovakia

* Corresponding author

e-mail: domenico.pangallo@savba.sk; Tel.: 00421 2 59307443

Table S1. List of band assignments according to Kansiz *et al.* (1999).

Wavenumber (cm^{-1})	Functional group(s)	Proposed band assignment
**1736**	ν C=O	Aliphatic aldehydes, conjugated carbonyl structures
**1406**	ν OC–OH	Carboxylic acid
**1362, 1319**	ν C–OH	Alcohols
**1286**	ν C–O	Carboxylic acid
**1231-900**	ν C–O–C	Polysaccharides

Kansiz, M. *et al.* Fourier Transform Infrared microspectroscopy and chemometrics as a tool for the discrimination of cyanobacterial strains. *Phytochemistry* **52**, 407-417 (1999).

Table S2. Bacterial and fungal clones detected on the albumen photographs and the album paper frame.

Sample of isolation	Clones identified based on the highest 16S and 28S rDNA similarity scores	Percentage of detection	Accession Number
**Gyula Albumen photograph**	**Bacteria**		
	JN871595 *Geobacillus thermoleovorans* 665/667 (99%)	35%	KT200278
	EU407552 *Bacillus pumilus* 657/660 (99%)	9%	KT200279
	CP000557 *Geobacillus thermodenitrificans* 662/666 (99%)	20%	KT200281
	CP006254 *Geobacillus* sp. 662/667 (99%)	6%	KT200282
	CP006579 *Aeromonas hydrophila* 657/659 (99%)	11%	KT200283
	KP229551 *Geobacillus* sp. 582/594 (98%)	13%	KT200284
	HQ757881 Uncultured bacterial clone 658/660 (99%) / NR_074931 *Geobacillus thermoleovorans* 657/660 (99%) [a]	6%	KT200285
	**Eukaryotes**		
	JQ807931 *Nectria* sp. 563/563 (100%)	31%	KT200297; KT200303
	KC425289 *Chaetomium globosum* 559/560 (99%)	10%	KT200298
	KM521834 *Cladosporium ramotenellum* 565/565 (100%)	16%	KT200299
	EU034632 *Saccharomyces servazzii* 562/572 (98%)	5%	KT200300
	AF075520 *Trichosporon aquatile* 596/596 (100%)	10%	KT200301
	FJ432593 *Geotrichum* sp. 540/545 (99%)	16%	KT200302
	AY464871 Uncultured fungus clone 564/573 (98%) / KJ170228 *Corynespora* sp. 562/572 (98%) [b]	2%	KT200304
	KJ847249 *Malassezia* sp. 598/599 (99%)	10%	KT200305
**Gyula Album paper frame**	**Bacteria**		
	CP006579 *Aeromonas hydrophila* 659/659 (100%)	11%	KT200286
	CP006254 *Geobacillus* sp. 659/667 (99%)	16%	KT200287
	CP010816 *Escherichia coli* 655/657 (99%)	4%	KT200288
	JN871595 *Geobacillus thermoleovorans* 665/667 (99%)	40%	KT200280
	KC252984 *Geobacillus kaustophilus* 664/667 (99%)	20%	KT200290

	GQ061009 Uncultured bacterium clone 633/633 (100%) / NR_042138 *Corynebacterium macginleyi* 630/633 (99%) [a]	9%	KT200291
	**Eukaryotes**		
	JQ807931 *Nectria* sp. 562/563 (99%)	49%	KT200306
	KJ847249 *Malassezia* sp. 599/599 (100%)	16%	KT200307
	HM014472 *Malassezia* sp. 595/598 (99%)	20%	KT200308
	JN974267 *Galactomyces candidum* 543/545 (99%)	15%	KT200309
**Antiquarian photograph**	**Bacteria**		
	JQ404482 *Shigella* sp. 628/629 (99%)	10%	KT200292
	AY550102 *Geobacillus thermoleovorans* 667/667 (100%)	13%	KT200289
	JN411418 *Staphylococcus succinus* 663/664 (99%)	10%	KT200293
	KM975639 *Lactococcus lactis* 661/663 (99%)	23%	KT200294
	KJ531396 *Lactococcus lactis* subsp. *cremoris* 661/663 (99%)	25%	KT200295
	KC252984 *Geobacillus kaustophilus* 663/667 (99%)	19%	KT200296
	**Eukaryotes**		
	EF652440 *Aspergillus* sp. 571/571 (100%)	19%	KT200310
	EF652088 *Eurotium halophilicum* 571/572 (99%)	22%	KT200311
	U81264 *Aspergillus penicillioides* 570/571 (99%)	19%	KT200312
	KC288123 *Alternaria* sp. 570/571 (99%)	10%	KT200313
	AY818962 *Gnomonia setacea* 562/562 (100%)	12%	KT200314
	KM458634 *Cladosporium* sp. 565/565 (100%)	8%	KT200315
	Z95383 *Trebouxia impressa* 561/565 (99%)	10%	KT200316

a) The BLAST search was also performed using the specific 16S rRNA database for bacteria and archaea.
b) The BLAST search was also performed excluding the Uncultured/environmental sample sequences.

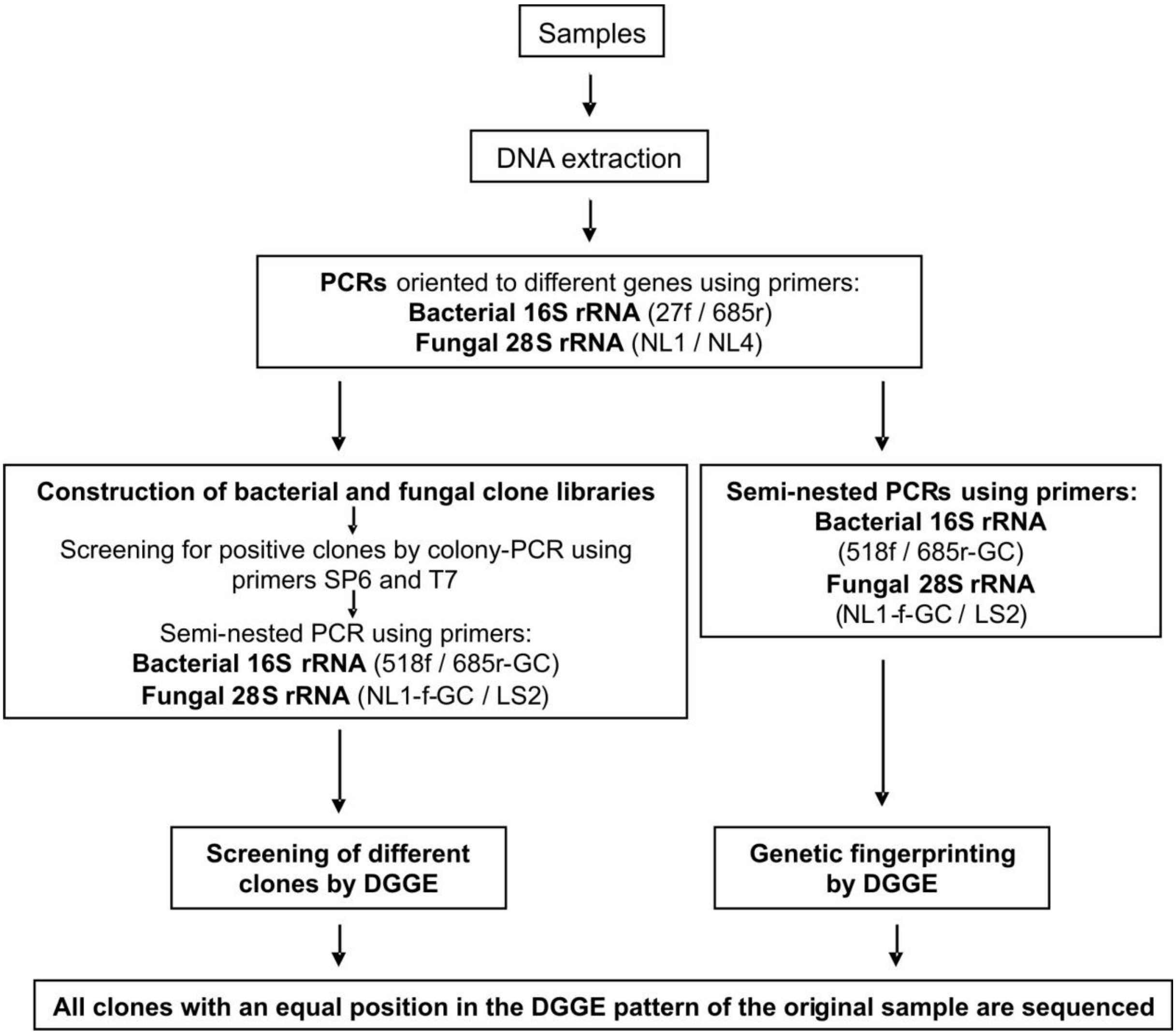

Fig. S1. Schematic diagram of the experimental procedure to analyze the microbial communities' diversity using the DGGE-cloning strategy.
